# Integrative Transcriptomic Analysis Reveals Distinct and Shared Host Responses in Dengue and Chikungunya Infections

**DOI:** 10.3390/ijms27125552

**Published:** 2026-06-19

**Authors:** Mostafa Rezapour, Thomas D. Shupe, David A. Ornelles, Sean V. Murphy, Anthony Atala

**Affiliations:** 1 Wake Forest Institute for Regenerative Medicine, Wake Forest University School of Medicine, Winston-Salem, NC 27101, USA; 2 Department of Microbiology and Immunology, Wake Forest University School of Medicine, Winston-Salem, NC 27101, USA

**Keywords:** dengue, chikungunya, transcriptomics, RNA sequencing, feature selection, consensus signatures, predictive modeling, interferon response

## Abstract

Dengue virus (DENV) and chikungunya virus (CHIKV) co-circulate in many regions and present with overlapping clinical features, which complicate accurate diagnosis and disease management. This study develops an integrative transcriptomic framework to identify robust host gene signatures that distinguish between dengue, chikungunya, and healthy states. Publicly available RNA sequencing (RNA-seq) datasets derived from human blood samples were analyzed using a cross-validation design to ensure robustness and prevent information leakage. Differential expression analysis was performed independently within each dataset using the Generalized Linear Models with Quasi-Likelihood F-tests and Magnitude–Altitude Scoring (GLMQL-MAS) framework, followed by Cross-Magnitude–Altitude Scoring (Cross-MAS) integration to identify shared and virus-specific gene signatures. A strict consensus approach across folds was applied to derive reproducible gene sets. These signatures were used for dimensionality reduction and multinomial logistic regression to evaluate classification performance. A small subset of selected genes showed strong discriminative performance within the cross-validation framework, with test balanced accuracy reaching 0.97, which improved upon models using all genes. Biologically, both infections exhibited a shared antiviral response characterized by interferon signaling and innate immune activation. However, distinct virus-specific patterns were identified. Dengue infection was associated with cell-cycle and DNA replication pathways, while chikungunya infection showed stronger enrichment of inflammatory and immune signaling pathways, including NF-kappaB and Toll-like receptor signaling. Overall, this study provides a cross-validation-based framework for integrative transcriptomic analysis and identifies compact, reproducible host-response signatures with strong discriminative signals in the analyzed cohorts. These signatures require validation in larger independent cohorts before any clinical or diagnostic application.

## 1. Introduction

Dengue virus (DENV) and chikungunya virus (CHIKV) are mosquito-borne viruses that cause dengue and chikungunya, respectively, and represent major global public health concerns, particularly in tropical and subtropical regions [1]. Despite their overlapping epidemiology and clinical presentation, the two viruses are taxonomically and biologically distinct: DENV is a flavivirus in the family Flaviviridae [2], whereas CHIKV is an alphavirus in the family Togaviridae [3]. These differences in viral family, genome organization, and replication biology may contribute to distinct host-response patterns after infection. Although both viruses often co-circulate and present with overlapping clinical symptoms, including fever, joint pain, and rash, they differ in disease progression, severity, and long-term outcomes [4,5,6,7,8]. Accurate differentiation between these infections is therefore essential for appropriate clinical management and epidemiological surveillance.

Host transcriptomic profiling has emerged as a valuable approach to characterize molecular responses to arboviral infections and to identify biomarkers associated with disease progression. In dengue, transcriptomic studies have identified gene signatures associated with severity, including a 20-gene panel predictive of progression to severe dengue across multiple cohorts [9], as well as individual transcripts such as *CFD*, *MAGED1*, and *PSMB9* that vary with disease severity [10].

In the case of chikungunya, transcriptomic insights have been used to understand the early molecular mechanisms that lead to post-acute and chronic musculoskeletal symptoms, which can persist for years [11]. A systems analysis of acute chikungunya infection identified distinct transcriptional changes correlating with viral load, symptom onset, and the activation of the NLRP3 inflammasome in macrophages [12]. The study highlighted the significant role of Eukaryotic Initiation Factor (eIF) family genes and *APOBEC3A* in the viral replication process [12].

Comparative studies have begun to explore shared and distinct host responses between DENV and CHIKV. Multi-omic analyses have demonstrated broadly similar innate immune activation during acute infection, alongside divergence in downstream immune pathways, including differences in interferon responses, monocyte activity, and adaptive immune profiles [13]. Cytokine-based studies have similarly identified both overlapping inflammatory signatures and virus-specific differences in cytokine expression and kinetics [14]. In addition, investigations in mosquito vectors have revealed both conserved antiviral pathways and virus-specific transcriptional responses [15], while cellular studies have highlighted asymmetric viral interactions and modulation of host antiviral signaling during coinfection [16].

Although these studies provide important insights, several limitations remain. First, many analyses focus on individual cohorts, specific molecular layers such as cytokines, or single-virus investigations, which limits the ability to directly compare host transcriptomic responses across infections. Second, existing comparative studies often emphasize descriptive differences rather than identifying robust, reproducible gene signatures that can distinguish between DENV and CHIKV infections. Third, there is a lack of integrative frameworks that combine rigorous feature selection, cross-dataset comparison, and predictive modeling while minimizing biases such as information leakage and dataset-specific effects.

**Figure 1:**
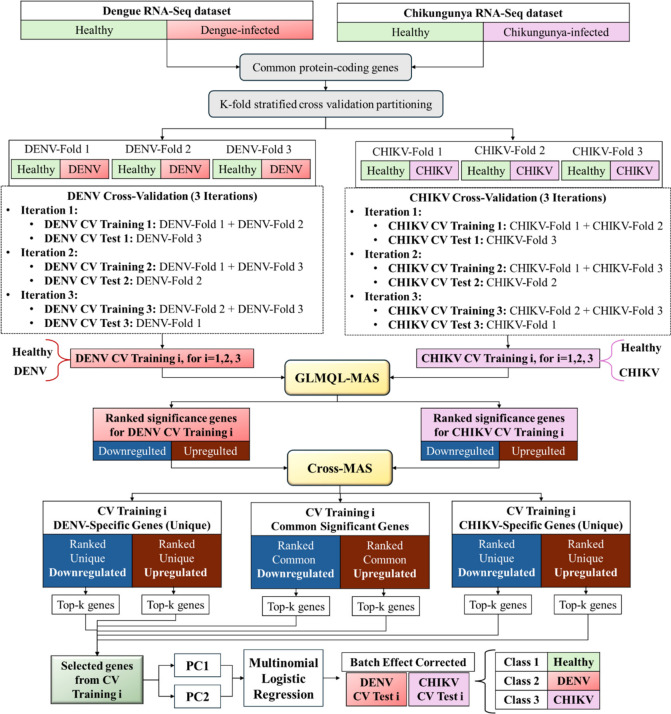
Overview of the analytical workflow for one cross-validation iteration. Each dataset (dengue and chikungunya) is first partitioned independently into stratified folds. Within each training fold, differential expression analysis is performed using Generalized Linear Models with Quasi-Likelihood F-tests and Magnitude–Altitude Scoring (GLMQL-MAS) to obtain ranked lists of upregulated and downregulated genes. Cross-Magnitude–Altitude Scoring (Cross-MAS) is then applied separately to integrate rankings across the two datasets, which produces six gene groups: common and virus-specific genes for each regulation direction. The top-*k* genes from each group are selected and used for dimensionality reduction via principal component analysis, followed by multinomial logistic regression to classify samples as healthy, dengue-infected, or chikungunya-infected. Model performance is evaluated on the corresponding independent test fold.

To address these gaps, the present study develops an integrative transcriptomic framework to systematically characterize shared and virus-specific host responses in dengue and chikungunya infections. The overall analytical workflow is summarized in Figure 1. Publicly available human blood-derived RNA sequencing (RNA-seq) datasets (GSE279208 and PRJNA507472) for dengue and chikungunya were first processed independently, and each dataset was partitioned into stratified cross-validation (CV) folds [17]. Within each training fold, differential expression analysis was performed separately for each virus by comparing infected samples with their corresponding healthy controls. Genes were then ranked using the Generalized Linear Models with Quasi-Likelihood F-tests and Magnitude–Altitude Scoring (GLMQL-MAS) [18,19,20,21,22,23,24], which integrates statistical significance and expression-change magnitude to prioritize infection-associated transcripts.

After fold-specific feature selection was completed independently in each dataset, matched dengue and chikungunya folds were aligned for cross-dataset integration. Cross-Magnitude–Altitude Scoring (Cross-MAS) [20] was then applied separately to upregulated and downregulated genes to identify shared antiviral signatures as well as dengue-specific and chikungunya-specific transcriptional patterns. The top-ranked genes from these categories were used for dimensionality reduction and multinomial logistic regression to evaluate whether compact gene panels could distinguish healthy, dengue-infected, and chikungunya-infected samples. Finally, strict consensus signatures were derived by retaining genes consistently identified across all training folds, and these reproducible gene sets were used for downstream functional enrichment analysis.

This design allowed predictive modeling and biological interpretation to be performed within a cross-validation framework while reducing information leakage and fold-specific bias.

## 2. Results

### 2.1. Fold-Specific Differential Expression Analysis Reveals Robust Transcriptional Signatures

**Figure 2:**
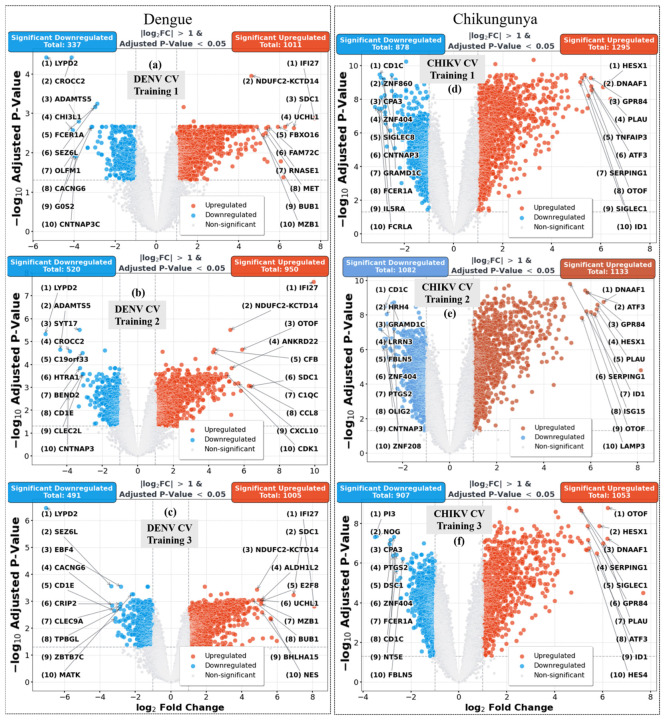
Fold-specific differential expression results for dengue and chikungunya across cross-validation training sets. Volcano plots show differential gene expression obtained using the GLMQL-MAS framework within each training partition (CV Training Sets 1–3). Panels (**a**–**c**) correspond to dengue virus (DENV) and panels (**d**–**f**) correspond to chikungunya virus (CHIKV). In each plot, genes are colored according to significance and direction of change: upregulated (red), downregulated (blue), and non-significant (gray), based on thresholds of adjusted *p*-value < 0.05 and *|log*_2_*FC|* > 1. The total number of significantly upregulated and downregulated genes is indicated in each panel. The top 10 upregulated and top 10 downregulated genes, ranked by Magnitude–Altitude Scoring (MAS) as defined in Equation (1), are annotated to highlight the most strongly associated features within each training fold. Due to marker size in the visualization, some genes may appear to lie exactly at *|log*_2_*FC|* = 1, although all reported significant genes satisfy the strict threshold *|log*_2_*FC|* > 1.

To characterize transcriptional responses associated with dengue and chikungunya infections, differential expression analysis was performed independently within each cross-validation training partition using the GLMQL-MAS framework [18,19,20,21,22,23,24]. Volcano plots summarizing the results for all three training folds are shown in Figure 2 for both viruses.

Across all training sets, a large number of genes satisfied the significance criteria (adjusted *p*-value < 0.05 and *|log*_2_*FC|* > 1), which confirmed the presence of strong infection-associated transcriptional signals in both datasets. For dengue (Figure 2a–c), the number of significantly upregulated genes ranged from 950 to 1011, while the number of downregulated genes ranged from 337 to 520 across the three folds. For chikungunya (Figure 2d–f), a larger number of differentially expressed genes was observed overall, with upregulated genes ranging from 1133 to 1295 and downregulated genes ranging from 878 to 1082. The complete fold-specific differential expression outputs are provided in Table S1.

The volcano plots demonstrated a consistent asymmetric distribution in both infections, which showed a predominance of upregulated genes relative to downregulated genes. This pattern was more pronounced in the chikungunya dataset, which exhibited a broader spread of highly significant upregulated genes across all folds.

To highlight the most biologically informative signals, the top 10 upregulated and top 10 downregulated genes ranked by Magnitude–Altitude Scoring (MAS) [25,26] (see Equation (1) in Section 4) were annotated in each plot. Several genes were consistently identified among the highest-ranked features across folds. In dengue, genes such as *IFI27*, *SDC1*, and *NDUFC2-KCTD14* were repeatedly among the top upregulated candidates, while *LYPD2* appeared among the most prominent downregulated genes. In chikungunya, strong and consistent upregulation was observed for genes including *HESX1*, *DNAAF1*, *GPR84*, and *PLAU*, whereas genes such as *CD1C* were frequently among the top downregulated features.

### 2.2. Cross-Dataset Integration Reveals Shared and Virus-Specific Gene Signatures

**Figure 3:**
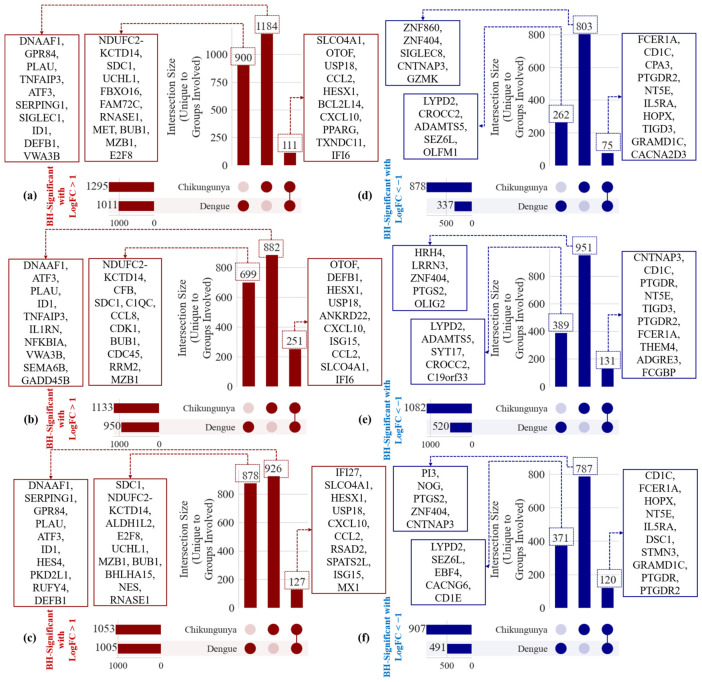
Cross-MAS integration of dengue and chikungunya gene signatures across cross-validation training folds. UpSet plots show the overlap and specificity of differentially expressed genes identified using the Cross-MAS ranking strategy (Equation (2)) within each aligned cross-validation training partition (CV Training Sets 1–3). Left panels (**a**–**c**) correspond to upregulated genes, and right panels (**d**–**f**) correspond to downregulated genes. For each fold, genes are partitioned into three categories: dengue-specific, chikungunya-specific, and shared (common) genes. Bar plots indicate the size of each intersection, which represents the number of genes uniquely belonging to each category or shared between datasets. The total number of significant genes for dengue and chikungunya within each fold is shown below each panel. For each category, the top-ranked genes based on Cross-MAS are listed to highlight the most informative features contributing to virus-specific and shared transcriptional responses.

Following fold-specific differential expression analysis, Cross-MAS was applied within each aligned cross-validation training partition to integrate dengue and chikungunya gene rankings as defined in Equation (2) in Section 4. The resulting UpSet plots for all three folds are shown in Figure 3, separately for upregulated and downregulated genes. The complete Cross-MAS ranked gene lists for each cross-validation fold are provided in Table S2.

Across all folds, Cross-MAS partitioned genes into three main categories within each regulation direction: dengue-specific, chikungunya-specific, and shared (common) genes. The UpSet plots revealed that a substantial number of genes were uniquely associated with each infection, while a smaller but consistent subset of genes was shared between dengue and chikungunya, which reflected common host-response mechanisms.

For upregulated genes, the largest intersections were consistently observed in the chikungunya-specific category across all folds, which is consistent with the broader transcriptional activation observed in Figure 2. In CV Training Set 1, 1184 genes were identified as chikungunya-specific, compared to 900 dengue-specific genes and 111 shared genes. Similar patterns were observed in CV Training Sets 2 and 3, where chikungunya-specific genes (882 and 926, respectively) exceeded dengue-specific genes (699 and 878), while the shared component remained relatively smaller (251 and 127 genes, respectively).

Inspection of the top-ranked genes within each category revealed consistent biological signals across folds. Chikungunya-specific upregulated genes included *DNAAF1*, *GPR84*, *PLAU*, *TNFAIP3*, *ATF3*, and *SERPING1*, which were repeatedly among the highest-ranked features. Dengue-specific upregulated genes were enriched for candidates such as *NDUFC2-KCTD14*, *SDC1*, *UCHL1*, and *RNASE1*. In contrast, the shared upregulated signature included genes such as *IFI27*, *USP18*, *HESX1*, *CXCL10*, *CCL2*, and *ISG15*, which suggested a common interferon-related response across both infections.

For downregulated genes, a similar structure was observed, although the overall number of genes was lower than for upregulation. In CV Training Set 1, 803 chikungunya-specific, 262 dengue-specific, and 75 shared downregulated genes were identified. This pattern persisted across CV Training Sets 2 and 3, where chikungunya-specific downregulated genes remained the largest group, followed by dengue-specific genes, with a smaller shared subset. Overall, the Cross-MAS integration demonstrated that, although dengue and chikungunya share a common core transcriptional response, each infection is characterized by a distinct set of strongly regulated genes.

### 2.3. Predictive Modeling Using Cross-MAS–Selected Gene Signatures Improves Classification Performance

To evaluate the discriminative power of the identified gene signatures, predictive modeling was performed within each cross-validation fold as described in Section 4.2.4. Briefly, the top-*k* genes from each Cross-MAS category (dengue-specific, chikungunya-specific, and shared; upregulated and downregulated) were selected within each training fold and used for dimensionality reduction via principal component analysis (PCA). The first two principal components (PC1 and PC2) were then used as predictors in a multinomial logistic regression model to classify samples as healthy, dengue-infected, or chikungunya-infected.

PCA was fit exclusively on the training data within each fold, where principal component axes were defined as linear combinations of the original gene expression features that capture directions of maximal variance in the training set. Test samples were subsequently projected onto these same axes by applying the transformation learned from the training data, thereby representing each test sample in the same reduced-dimensional space without recomputing the PCA. This approach ensures that both training and test samples are expressed in a common feature space while preventing information from the test data from influencing the dimensionality reduction step.

**Figure 4:**
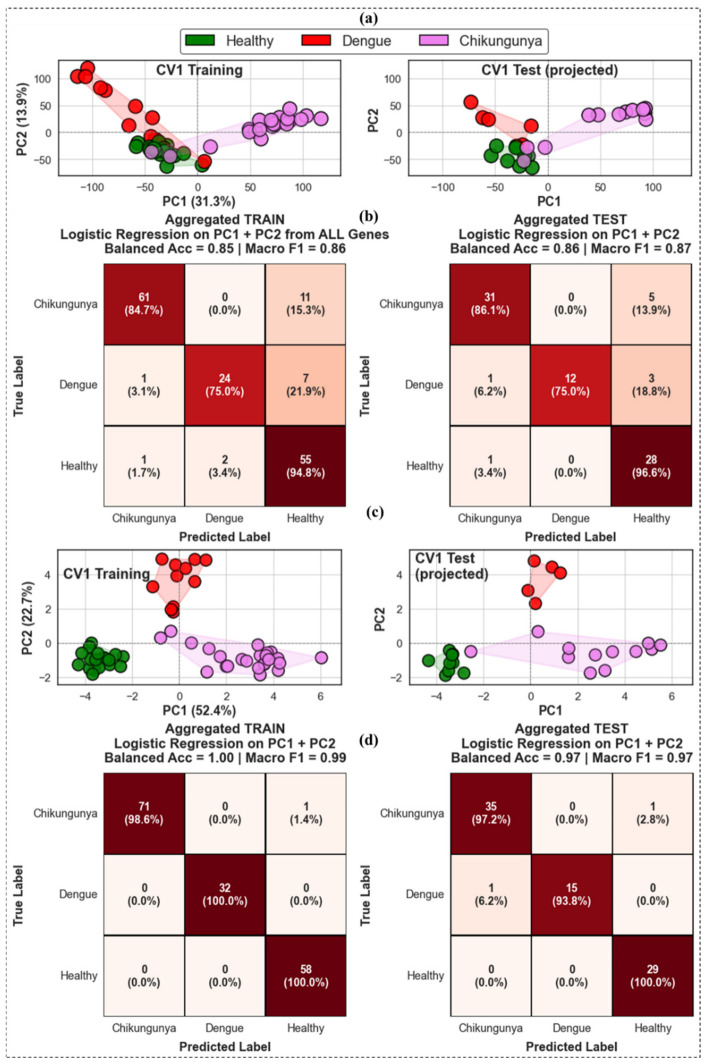
Predictive modeling performance using all genes versus Cross-MAS-selected gene signatures across cross-validation folds. (**a**) Principal component analysis (PCA) of CV Training Set 1 and projection of CV Test Set 1 using all shared protein-coding genes. PCA was fit on the training set only, and the test set was projected onto the same principal component axes derived from the training data. Thus, PC1 and PC2 in both panels represent a shared feature space. The first two principal components were used as input features for multinomial logistic regression. (**b**) Aggregated confusion matrices across all cross-validation training and test partitions using all genes, with corresponding balanced accuracy and macro F1 scores. (**c**) PCA of CV Training Set 1 and projection of CV Test Set 1 using the selected gene signatures, defined as the top *k* = 3 genes from each Cross-MAS category (dengue-specific, chikungunya-specific, and shared; upregulated and downregulated) within each training fold. Here, PCA was also fit on the training set only, and the test set was projected onto the training-derived PC axes. PC1 and PC2 were used as inputs for multinomial logistic regression. (**d**) Aggregated confusion matrices across all cross-validation training and test partitions using the selected genes and their corresponding PC1 and PC2 representations for multinomial logistic regression. Classification performance metrics are reported for both training and test data. In subfigures (**b**,**d**), darker colors in the confusion matrices indicate higher sample counts within each cell.

Model selection within each training fold indicated that *k* = 3, corresponding to the top three genes from each of the six categories (18 genes in total), provided the minimum number of features required to achieve optimal class separation. This value was therefore used consistently across all folds for downstream evaluation (see Figure 4).

To assess the impact of feature selection, classification performance was first evaluated using all protein-coding genes. As shown in Figure 4a, PCA projections for CV Training Set 1 and CV Test Set 1 demonstrated partial separation between the three classes, with noticeable overlap between dengue and chikungunya samples. The corresponding aggregated confusion matrices across all training and test folds (Figure 4b) showed moderate classification performance, with balanced accuracy of 0.85 (training) and 0.86 (test), and macro F1-scores of 0.86 and 0.87, respectively.

In contrast, when the analysis was restricted to the selected gene signatures (top 3 genes per category), a marked improvement in class separation was observed. PCA projections for CV Training Set 1 and CV Test Set 1 (Figure 4c) showed clear clustering of healthy, dengue, and chikungunya samples, with minimal overlap between classes. This improved separation was associated with strong internal classification performance across the cross-validation folds, as summarized by the aggregated confusion matrices. As shown in the aggregated confusion matrices (Figure 4d), the model achieved strong apparent performance on the training data (balanced accuracy = 1.00, macro F1 = 0.99) and high cross-validated test performance (balanced accuracy = 0.97, macro F1 = 0.97).

Therefore, misclassification rates were greatly reduced when using the selected gene sets, particularly between dengue and chikungunya samples. These results suggest that the Cross-MAS-derived gene signatures capture highly informative features for distinguishing between infections in the analyzed datasets. The consistency of performance across training and test partitions supports the internal robustness of the selected gene signatures, although external validation in larger cohorts will be needed to establish their generalizability.

**Figure 5:**
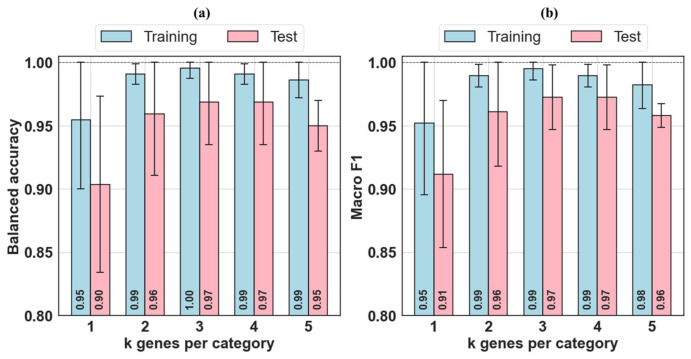
Sensitivity analysis of model performance across different *k* values. Classification performance was evaluated using the top-*k* genes per Cross-MAS category, with *k* ranging from 1 to 5. For each *k* value, the selected genes from the six Cross-MAS categories were used for PCA, and the first two principal components were used as predictors in a multinomial logistic regression model. Bars show the mean performance across the three cross-validation folds for training and independent test partitions. Error bars indicate the standard deviation across folds. Performance improved from *k* = 1 and remained high for *k* = 2 to *k* = 5, with *k* = 3 achieving the highest or near-highest test (**a**) balanced accuracy and (**b**) macro F1 while retaining a compact selected-gene panel.

To evaluate whether the selected value of *k* was sensitive to small changes in the number of genes retained per category, we additionally assessed model performance across *k* values from 1 to 5 genes per category. As shown in Figure 5, performance improved from *k* = 1 to *k* = 2 and remained consistently high for nearby values of *k*. The highest or near-highest test performance was observed at *k* = 3, with mean test balanced accuracy and macro F1 both reaching 0.97 across cross-validation folds. Similar performance was observed for *k* = 4, while *k* = 5 showed a slight decrease in test balanced accuracy. These results indicate that the model was not dependent on a single arbitrary *k* value, and that *k* = 3 provided a compact feature set with optimal or near-optimal predictive performance.

**Figure 6:**
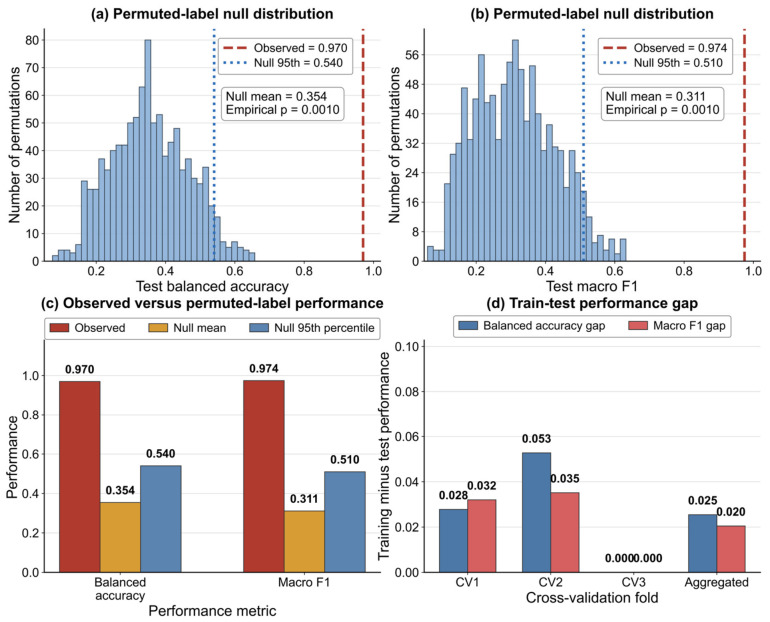
Label-permutation and train–test gap analyses for overfitting assessment. (**a**) Permuted-label null distribution for test balanced accuracy across 1000 permutations. The dashed red line indicates the observed true-label performance, and the dotted blue line indicates the 95th percentile of the permuted-label null distribution. (**b**) Permuted-label null distribution for test macro F1 across 1000 permutations. (**c**) Comparison of observed performance with the permuted-label null mean and 95th percentile for balanced accuracy and macro F1. (**d**) Train–test performance gap across cross-validation folds and the aggregated analysis, calculated as training performance minus test performance. The results did not show evidence that the observed performance was driven by random label structure or a large train–test discrepancy within the cross-validation framework.

To further assess potential overfitting, we performed a label-permutation analysis using the same selected-gene PCA and multinomial logistic regression workflow. Class labels were randomly permuted, and the modeling procedure was repeated for 1000 permutations to generate null distributions for test balanced accuracy and macro F1. As shown in Figure 6a,b, the observed true-label performance was far above the corresponding permuted-label null distributions. The observed test balanced accuracy was 0.970, compared with a permuted-label mean of 0.354 and a 95th percentile of 0.540. Similarly, the observed test macro F1 was 0.974, compared with a permuted-label mean of 0.311 and a 95th percentile of 0.510. None of the 1000 permuted-label models reached the observed true-label performance, yielding empirical *p*-values of 0.001 for both metrics. Direct comparison of observed performance with the permuted-label null mean and 95th percentile further confirmed that the true-label model substantially exceeded the null expectation (Figure 6c).

In addition, the train–test performance gap was small across the aggregated cross-validation analysis, with gaps of 0.025 for balanced accuracy and 0.020 for macro F1 (Figure 6d). Together, these results did not show evidence that the observed performance was driven by random class-label structure or a large train–test discrepancy within the cross-validation framework. The complete label-permutation and train–test gap results are provided in Table S6.

### 2.4. Consensus Gene Signatures Identify Robust Virus-Specific and Shared Transcriptional Responses

**Figure 7:**
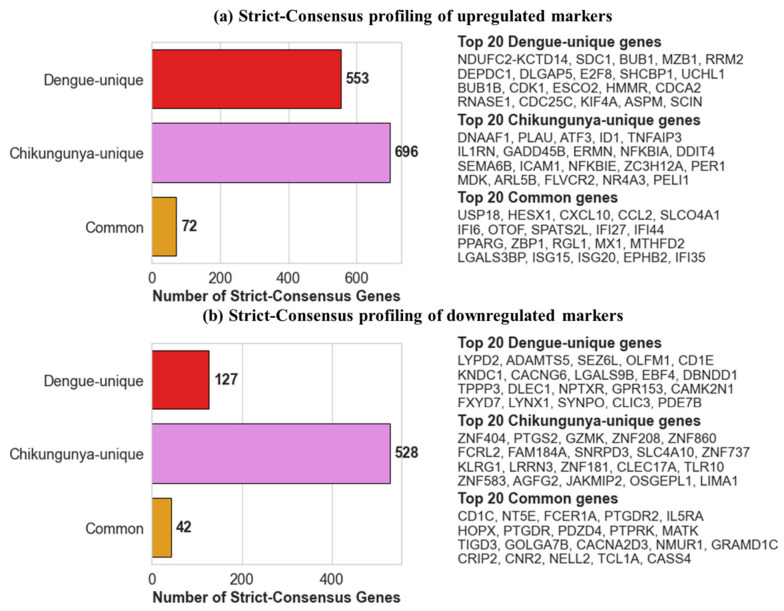
Strict consensus gene signatures across cross-validation folds for dengue and chikungunya infections. (**a**) Upregulated genes and (**b**) downregulated genes retained under the strict consensus criterion (intersection across all three training folds) for each Cross-MAS category: dengue-specific, chikungunya-specific, and shared (common) genes. Bar plots show the number of consensus genes in each category, with values indicated on the bars. For each category and regulation direction, the top 20 genes ranked by consensus rank are listed to highlight the most robust and reproducible features across folds.

To identify stable and reproducible gene signatures across data partitions, a strict consensus analysis was performed as described in Section 4.2.5 by retaining only genes consistently selected across all three training folds within each Cross-MAS category. The resulting consensus gene sets for upregulated and downregulated genes are summarized in Figure 7.

For upregulated genes, a total of 696 chikungunya-specific genes, 553 dengue-specific genes, and 72 shared genes were identified under the strict consensus criterion. This distribution indicates that chikungunya infection induces a broader and more consistent upregulated transcriptional response compared to dengue, while a smaller subset of genes is commonly activated in both infections. Among dengue-specific upregulated genes, top-ranked candidates included *NDUFC2-KCTD14*, *SDC1*, *BUB1*, *MZB1*, and *RRM2*, which are associated with cell-cycle regulation and cellular proliferation. In contrast, chikungunya-specific upregulated genes were enriched for inflammatory and stress-response regulators such as *DNAAF1*, *PLAU*, *ATF3*, *ID1*, and *TNFAIP3*. The shared upregulated signature included well-known interferon-stimulated and immune-response genes such as *USP18*, *HESX1*, *CXCL10*, *CCL2*, and *IFI6*, which suggests a common antiviral response across both infections. For downregulated genes, fewer consensus genes were identified overall, with 528 chikungunya-specific genes, 127 dengue-specific genes, and 42 shared genes. Similar to the upregulated patterns, chikungunya exhibited a larger and more consistent downregulated gene set compared to dengue. The complete strict consensus gene signatures are provided in Table S3.

To provide biological context for representative consensus genes, Table 1 summarizes their signature category and literature-supported relevance to antiviral, inflammatory, cell-cycle, B-cell/plasmablast, endothelial, myeloid/macrophage, and complement-related processes.

To further illustrate the discriminative power of the consensus gene signatures, PCA and multinomial logistic regression were applied to the entire dataset using either all protein-coding genes or the top-ranked consensus genes. These analyses were performed for visualization and interpretability purposes only and not for predictive evaluation, as model performance had already been assessed using cross-validation in Section 2.3.

**Figure 8:**
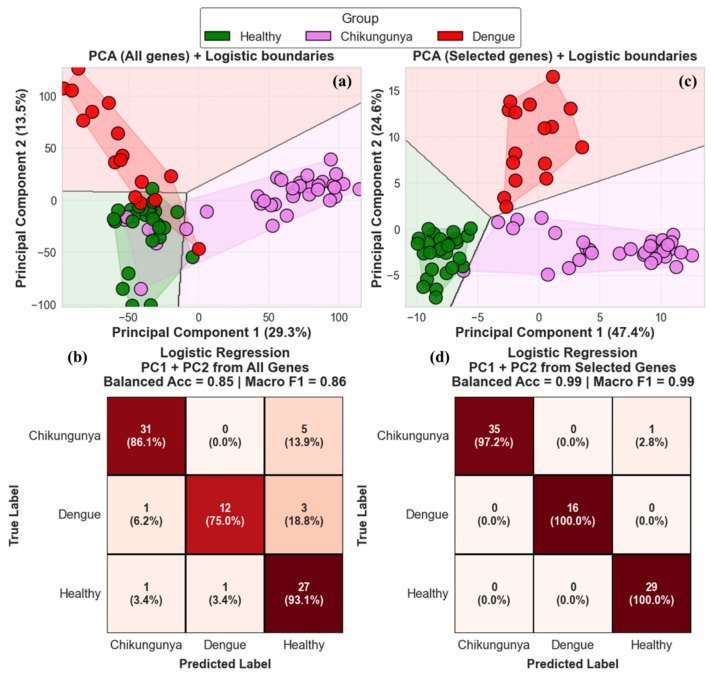
Global separation of sample groups using all genes versus consensus gene signatures. (**a**) PCA projection of all samples using all protein-coding genes. (**b**) PCA projection using the top 20 consensus genes from each category (dengue-specific, chikungunya-specific, and shared). In panels (**a**,**b**), the lines represent the decision boundaries from the logistic regression model, and the colored regions indicate the predicted class for each area of the PCA space. (**c**) Confusion matrix obtained by multinomial logistic regression using PC1 and PC2 derived from all genes. (**d**) Confusion matrix obtained using PC1 and PC2 derived from the selected consensus genes. In subfigures (**b**,**d**), darker colors in the confusion matrices indicate higher sample counts within each cell.

As shown in Figure 8a, PCA based on all protein-coding genes resulted in partial separation between healthy, dengue, and chikungunya samples, with noticeable overlap between dengue and chikungunya clusters. In contrast, when PCA was performed using only the top 20 consensus genes from each category (Figure 8b), a clear and well-defined separation between the three groups emerged, with minimal overlap between clusters. This result indicates that the consensus gene signatures capture the most informative axes of variation relevant to infection status.

To further visualize class separability, multinomial logistic regression models were fitted using PC1 and PC2 derived from each feature set. The resulting confusion matrices are shown in Figure 8c,d. When all genes were used (Figure 8c), classification performance was moderate. In contrast, when using the selected consensus genes (Figure 8d), very high classification performance was observed, with balanced accuracy and macro F1-scores of 0.99, and no misclassification of healthy or dengue samples. Together, these findings suggest that the selected consensus genes provide a compact and highly informative representation of host transcriptional responses, with strong ability to distinguish between healthy, dengue, and chikungunya samples in the analyzed cohorts.

### 2.5. Functional Enrichment Analysis

**Figure 9:**
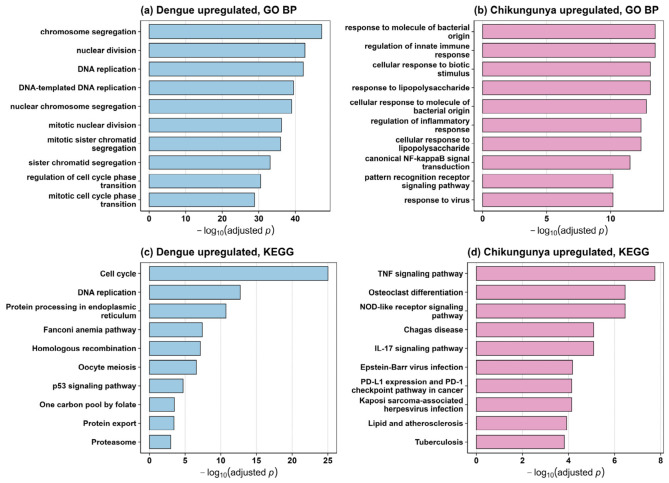
Virus-level functional enrichment. For each infection, virus-level upregulated gene sets were analyzed to compare dominant functional patterns before shared and virus-specific interpretation. (**a**) Top 10 Gene Ontology Biological Process (GO BP) terms for dengue upregulated genes. (**b**) Top 10 GO BP terms for chikungunya upregulated genes. (**c**) Top 10 Kyoto Encyclopedia of Genes and Genomes (KEGG) pathways for dengue upregulated genes. (**d**) Top 10 KEGG pathways for chikungunya upregulated genes. Bar lengths represent −log10(adjusted *p*-value).

We first performed virus-level enrichment analysis to evaluate the dominant biological themes within each infection. For upregulated genes, dengue showed strong enrichment of cell-cycle and DNA-replication terms, including chromosome segregation, nuclear division, DNA replication, and mitotic cell-cycle phase transition (Figure 9a). Kyoto Encyclopedia of Genes and Genomes (KEGG) analysis similarly highlighted Cell cycle, DNA replication, Homologous recombination, Fanconi anemia pathway, and p53 signaling pathway (Figure 9c). In contrast, chikungunya showed enrichment of innate immune and inflammatory processes, including regulation of innate immune response, response to lipopolysaccharide, canonical NF-kappaB signal transduction, pattern recognition receptor signaling pathway, and response to virus (Figure 9b). KEGG pathways enriched in chikungunya included TNF signaling pathway, NOD-like receptor signaling pathway, IL-17 signaling pathway, and infection-associated immune pathways (Figure 9d). The complete virus-level enrichment outputs for both upregulated and downregulated gene sets are provided in Table S4.

#### 2.5.1. Virus-Common Consensus Genes

**Figure 10:**
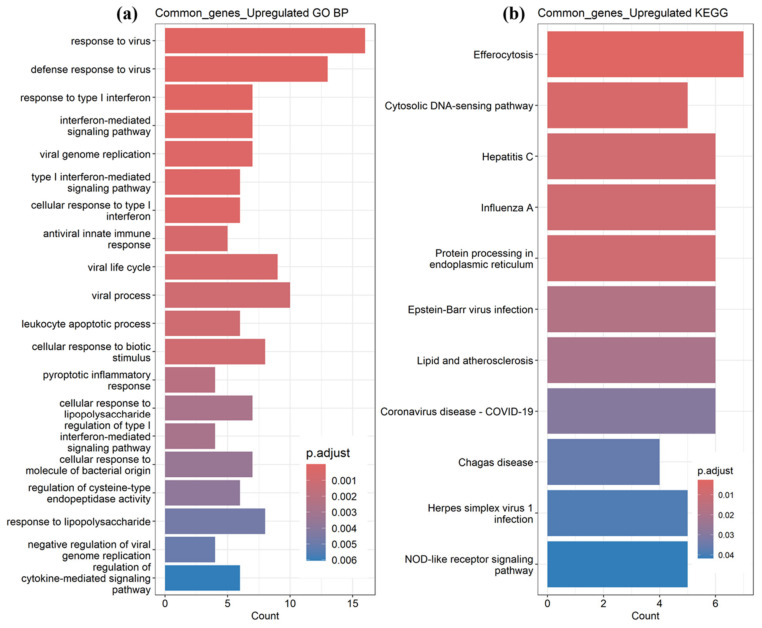
Functional enrichment analysis of upregulated shared consensus genes. (**a**) Gene Ontology Biological Process (GO BP) enrichment and (**b**) KEGG pathway enrichment for the upregulated common consensus gene set. Bars represent the number of genes associated with each term or pathway, and color indicates adjusted *p*-values. Enriched terms highlight antiviral responses, interferon signaling, and innate immune pathways shared between dengue and chikungunya infections.

To characterize the biological functions associated with the consensus gene signatures, functional enrichment analysis was performed using Gene Ontology Biological Process (GO BP) terms and KEGG pathways, as described in Section 4.2.6. The enrichment results for the upregulated shared (common) consensus genes are shown in Figure 10.

GO BP analysis (Figure 10a) showed enrichment of antiviral and immune-related processes. The most significant terms included “response to virus,” “defense response to virus,” and “response to type I interferon.” Additional enriched processes included “interferon-mediated signaling pathway,” “antiviral innate immune response,” “viral genome replication,” “leukocyte apoptotic process,” “pyroptotic inflammatory response,” and “cytokine-mediated signaling pathway.”

Consistent with these findings, KEGG pathway analysis (Figure 10b) identified enrichment of pathways related to viral infection and innate immune signaling. Prominent pathways included “Cytosolic DNA-sensing pathway,” “NOD-like receptor signaling pathway,” and “Protein processing in endoplasmic reticulum.” Several virus-related pathways, including “Influenza A,” “Hepatitis C,” “Epstein-Barr virus infection,” and “Coronavirus disease (COVID-19),” were also significantly enriched.

#### 2.5.2. Virus-Specific Consensus Genes

**Figure 11:**
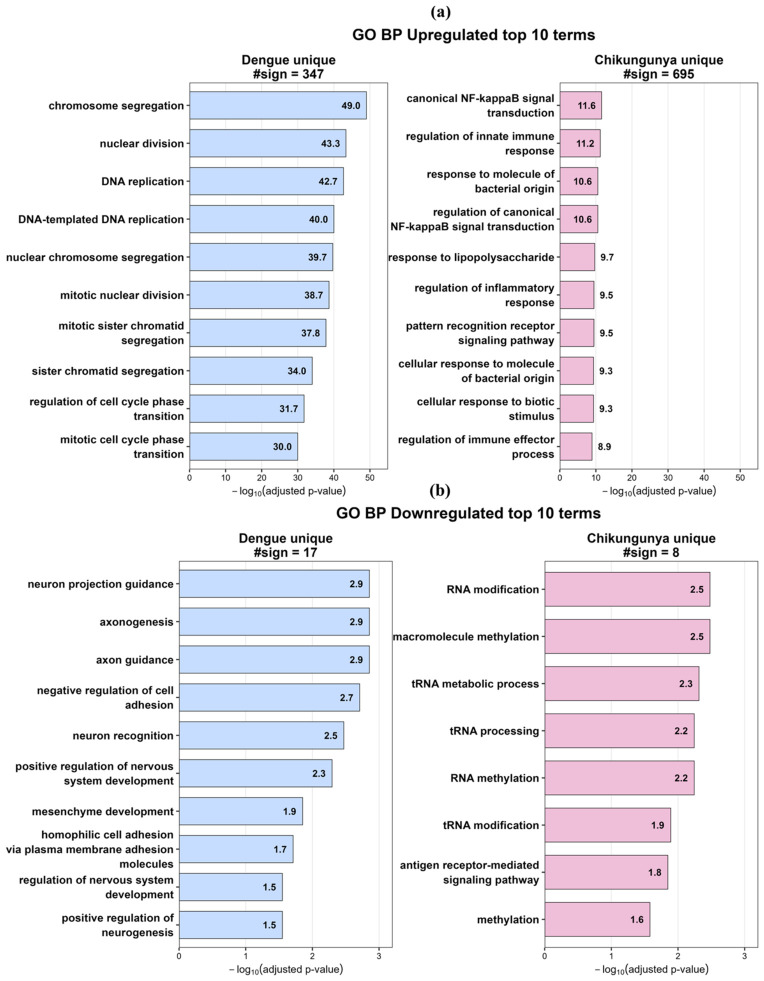
Functional enrichment of virus-specific consensus genes. (**a**) Top 10 enriched GO Biological Process (GO BP) terms for upregulated dengue-specific (**left**) and chikungunya-specific (**right**) consensus genes. (**b**) Top 10 enriched GO BP terms for downregulated dengue-specific (**left**) and chikungunya-specific (**right**) consensus genes. Bar lengths represent −log10(adjusted *p*-value), and the number of significant genes in each category is indicated above each panel.

To further characterize infection-specific biological processes, functional enrichment analysis was performed on dengue-specific and chikungunya-specific consensus genes separately for upregulated and downregulated signatures. The top enriched GO BP terms are shown in Figure 11.

For upregulated genes, dengue-specific consensus genes (Figure 11a, left) were strongly enriched for cell-cycle-related processes, including “chromosome segregation,” “nuclear division,” “DNA replication,” and “mitotic cell cycle phase transition.” In contrast, chikungunya-specific upregulated genes (Figure 11a, right) were enriched for immune and inflammatory pathways, including “canonical NF-kappaB signal transduction,” “regulation of innate immune response,” “response to lipopolysaccharide,” and “pattern recognition receptor signaling pathway.”

For downregulated genes, fewer significant terms were identified in both infections. Dengue-specific downregulated genes (Figure 11b, left) were enriched for processes related to neuronal development and cell adhesion, including “neuron projection guidance,” “axonogenesis,” and “cell adhesion.” Chikungunya-specific downregulated genes (Figure 11b, right) were primarily associated with RNA-related processes, including “RNA modification,” “tRNA metabolic process,” “RNA methylation,” and “macromolecule methylation.”

**Figure 12:**
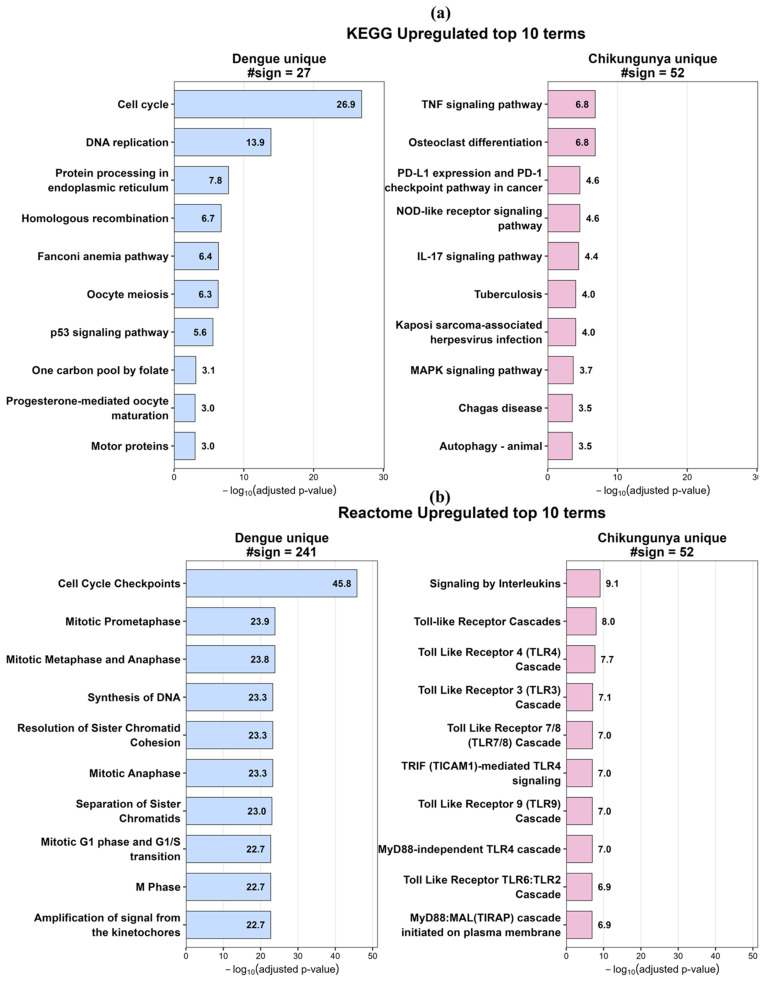
KEGG and Reactome enrichment of virus-specific upregulated consensus genes. (**a**) Top 10 enriched KEGG pathways and (**b**) top 10 enriched Reactome pathways for dengue-specific (**left**) and chikungunya-specific (**right**) upregulated consensus genes. Bar lengths represent −log10(adjusted *p*-value), and the number of significant genes is indicated above each panel.

To further evaluate pathway-level differences between dengue and chikungunya infections, KEGG and Reactome enrichment analyses were performed on virus-specific upregulated consensus genes. The top enriched pathways are shown in Figure 12.

KEGG pathway analysis (Figure 12a) showed distinct functional profiles between the two infections. Dengue-specific upregulated genes were primarily enriched in pathways related to cell-cycle regulation and DNA replication, including “Cell cycle,” “DNA replication,” and “Homologous recombination.” Additional enriched pathways included “p53 signaling pathway” and “Fanconi anemia pathway.” In contrast, chikungunya-specific upregulated genes were enriched in immune and inflammatory signaling pathways, including “TNF signaling pathway,” “NOD-like receptor signaling pathway,” and “IL-17 signaling pathway,” as well as infection-related pathways such as “Tuberculosis” and “Kaposi sarcoma-associated herpesvirus infection.”

Reactome pathway analysis (Figure 12b) showed enrichment of cell-cycle-related processes in dengue, including “Cell Cycle Checkpoints,” “Mitotic Prometaphase,” and “Synthesis of DNA.” In contrast, chikungunya-specific genes were enriched for immune signaling cascades, particularly Toll-like receptor-mediated pathways, including “Toll-like Receptor Cascades,” “TLR4 Cascade,” “TLR3 Cascade,” and “TLR7/8 Cascade,” as well as “Signaling by Interleukins.” The complete shared and virus-specific enrichment outputs for both upregulated and downregulated gene sets are provided in Table S5.

## 3. Discussion

In this study, we developed an integrative transcriptomic framework to evaluate predictive modeling performance and identify gene signatures that distinguish dengue and chikungunya infections. Using a cross-validation-based design, differential expression analysis was first performed independently within each dataset, followed by Cross-MAS integration to define shared and virus-specific gene groups. These gene sets were then used for predictive modeling, and a strict consensus approach was applied to derive robust gene signatures for downstream biological interpretation.

The predictive modeling results suggest that the selected gene signatures provide strong discriminative performance within the analyzed cohorts. While models based on all protein-coding genes showed only moderate separation between dengue and chikungunya, the use of a small subset of Cross-MAS-selected genes substantially improved classification performance. In particular, the selected genes yielded strong separation between healthy, dengue, and chikungunya samples across cross-validation folds, with high performance maintained in the independent test partitions. These findings suggest that the proposed feature selection strategy can capture highly informative transcriptional signals while reducing noise and dimensionality in these datasets.

These findings should not be interpreted as evidence that the proposed host-transcriptomic framework can replace standard diagnostic approaches. In clinical practice, dengue and chikungunya are commonly evaluated using molecular and serological assays, including reverse transcription polymerase chain reaction (RT-PCR), antigen detection, and enzyme-linked immunosorbent assay (ELISA)-based serology. In addition, if RNA-seq data were available for a clinical sample, viral reads could also be directly examined. Therefore, the gene signatures identified here should be interpreted as host-response signatures with discriminative signals in the analyzed datasets, rather than as a validated diagnostic assay. Their potential clinical utility would require validation in larger independent cohorts and comparison with established diagnostic methods.

Beyond predictive performance, the derivation of strict consensus gene signatures allowed for a more stable and interpretable characterization of host responses. By focusing on genes consistently identified across all training folds, the analysis reduced variability associated with individual data partitions and highlighted reproducible biological patterns. The strict consensus approach substantially reduced the gene sets to those consistently identified across folds, which enhances robustness and minimizes the influence of fold-specific variability.

A central observation across all analyses is the presence of a strong shared antiviral response between dengue and chikungunya. The common upregulated consensus genes were consistently enriched for interferon-related pathways and innate immune processes, including response to virus, type I interferon signaling, and antiviral innate immune response. The proposed framework did not simply recover a broad interferon-associated transcriptional response; rather, it distilled this shared response into a compact and reproducible gene signature with strong discriminatory value. The shared upregulated signature included interferon-stimulated and immune-response genes such as *USP18*, *HESX1*, *CXCL10*, *CCL2*, and *IFI6*, which suggests a common antiviral response across both infections. The GO BP enrichment terms “response to virus,” “defense response to virus,” and “response to type I interferon” indicate a robust activation of host antiviral defense mechanisms. Additional terms, including “interferon-mediated signaling pathway,” “antiviral innate immune response,” and “viral genome replication,” further support the central role of interferon-driven responses in both dengue and chikungunya infections.

The enrichment of pathways associated with multiple viral infections further supports the idea that this shared signature reflects a general antiviral state rather than virus-specific activity. This is expected, as early host responses to viral infection are often mediated by broadly acting innate immune pathways. Enrichment of terms such as “leukocyte apoptotic process,” “pyroptotic inflammatory response,” and “cytokine-mediated signaling pathway” also suggests that immune cell regulation and inflammatory responses are important components of the shared transcriptional program.

At the pathway level, the shared upregulated consensus genes were enriched for KEGG pathways related to viral infection and innate immune signaling. Prominent pathways included “Cytosolic DNA-sensing pathway,” “NOD-like receptor signaling pathway,” and “Protein processing in endoplasmic reticulum,” which are involved in pathogen recognition and antiviral responses. Several virus-related pathways, including “Influenza A,” “Hepatitis C,” “Epstein-Barr virus infection,” and “Coronavirus disease (COVID-19),” were also significantly enriched, which reflect common host-response mechanisms activated across diverse viral infections. Overall, these results suggest that the shared upregulated consensus genes capture a core antiviral transcriptional program dominated by interferon signaling, innate immune activation, and inflammatory responses, which are consistently observed in both dengue and chikungunya infections.

Several of the representative consensus genes identified in this study have also been reported in previous viral-infection and arbovirus studies, which provides additional biological support for the observed signatures. For example, the shared genes *USP18*, *ISG15*, *IFI27*, *CXCL10*, and *CCL2* are consistent with published interferon-stimulated and chemokine-associated antiviral responses. Among dengue-associated genes, *SDC1* and *MZB1* have been reported in dengue transcriptomic or disease-severity contexts, while *BUB1* and *RRM2* are consistent with the cell-cycle and DNA-replication patterns observed in the enrichment analysis. For chikungunya, genes such as *TNFAIP3*, *ATF3*, *GPR84*, and *SERPING1* support an inflammatory, stress-response, myeloid/macrophage-associated, and complement-regulatory interpretation.

Despite this shared response, the results demonstrate clear divergence in virus-specific transcriptional programs. Dengue-specific upregulated genes were strongly associated with cell-cycle-related processes, including DNA replication, chromosome segregation, and mitotic progression. Among dengue-specific upregulated genes, top-ranked candidates included *NDUFC2-KCTD14*, *SDC1*, *BUB1*, *MZB1*, and *RRM2*, which are associated with cell-cycle regulation and cellular proliferation. The GO BP enrichment results indicate that dengue infection is associated with cellular proliferation and DNA replication machinery. KEGG enrichment in “Cell cycle,” “DNA replication,” “Homologous recombination,” “p53 signaling pathway,” and “Fanconi anemia pathway” further supports the involvement of DNA damage response and cell-cycle control mechanisms.

Reactome enrichment in “Cell Cycle Checkpoints,” “Mitotic Prometaphase,” and “Synthesis of DNA” also indicates activation of proliferation-associated pathways. This pattern suggests that dengue infection may be linked to modulation of host cell proliferation and DNA replication machinery. One possible interpretation is that dengue virus may interact with host cell-cycle processes to facilitate viral replication or persistence. This interpretation should be considered hypothesis-generating, particularly because the present study was based on transcriptomic associations rather than direct experimental testing of viral manipulation of cell-cycle processes.

In contrast, chikungunya-specific upregulated genes were predominantly enriched for immune and inflammatory pathways, including NF-kappaB signaling, Toll-like receptor signaling, and cytokine-mediated responses. Chikungunya-specific upregulated genes were enriched for inflammatory and stress-response regulators such as *DNAAF1*, *PLAU*, *ATF3*, *ID1*, and *TNFAIP3*. GO BP terms including “canonical NF-kappaB signal transduction,” “regulation of innate immune response,” “response to lipopolysaccharide,” and “pattern recognition receptor signaling pathway” highlight strong activation of innate immune and inflammatory signaling pathways in chikungunya infection.

KEGG pathways such as “TNF signaling pathway,” “NOD-like receptor signaling pathway,” and “IL-17 signaling pathway,” together with Reactome pathways such as “Toll-like Receptor Cascades,” “TLR4 Cascade,” “TLR3 Cascade,” “TLR7/8 Cascade,” and “Signaling by Interleukins,” reflect activation of host defense mechanisms and inflammatory signaling. These pathways are central to innate immune activation and inflammatory signaling, which suggests that chikungunya infection induces a more pronounced inflammatory response compared to dengue in the analyzed cohorts. The enrichment of pathways such as TNF signaling and IL-17 signaling further supports this observation and may relate to the strong inflammatory symptoms commonly associated with chikungunya infection. Together, these findings indicate that while dengue is more associated with modulation of cellular processes, chikungunya is characterized by heightened immune activation.

The downregulated gene signatures also provide insight into infection-specific effects. Dengue-specific downregulated genes were enriched for processes annotated to neuronal development and cell adhesion, including “neuron projection guidance,” “axonogenesis,” and “cell adhesion.” Related neuronal-development or axon-associated annotations have been reported in DENV-associated omics contexts. For example, a comparative transcriptomic study in human neural progenitor cells reported that DENV-specific differentially expressed genes, particularly downregulated genes, were enriched in neuron differentiation, brain development, and Wnt signaling-related pathways [43]. In addition, a DENV central nervous system infection model reported downregulation of axonogenesis- and synapse-associated pathways during infection progression [44]. A DENV-associated peripheral blood mononuclear cell microRNA study also identified predicted target pathways including neurotrophin signaling and axon guidance [45]. Thus, the neuronal-development and axon-associated enrichment observed in the present blood-derived analysis is consistent with pathway-level patterns reported in other DENV-associated omics studies. Given the blood-derived nature of the present data, we interpret this finding as a transcriptomic pathway signal that may reflect shared biological programs related to cell adhesion, migration, cytoskeletal organization, immune regulation, or blood cell composition during infection, rather than as a direct measure of neurological involvement.

In contrast, chikungunya-specific downregulated genes were associated with RNA-related processes, including RNA modification and tRNA metabolism. Chikungunya-specific downregulated genes included *ZNF404*, *PTGS2*, *GZMK*, *ZNF208*, and *ZNF860*, and their association with “RNA modification,” “tRNA metabolic process,” “RNA methylation,” and “macromolecule methylation” suggests alterations in RNA processing and post-transcriptional regulation during infection. The shared downregulated genes included *CD1C*, *NT5E*, *FCER1A*, *PTGDR2*, and *IL5RA*. These shared downregulated genes may reflect common suppression of specific immune cell functions during infection. Because these observations are based on enrichment analysis, they should be interpreted as candidate biological patterns that require further validation.

The strong classification performance achieved using a small set of consensus genes further supports the potential biological relevance of these signatures. The observation that a limited number of genes separated healthy, dengue, and chikungunya samples in these cohorts suggests that the identified gene sets may capture major axes of variation associated with infection status. When PCA was performed using only the top 20 consensus genes from each category, a clear separation between the three groups emerged, which indicates that the consensus gene signatures capture informative axes of variation relevant to infection status. Together, these findings suggest that the selected consensus genes may provide a compact and highly informative representation of host transcriptional responses, with strong potential value to distinguish between healthy, dengue, and chikungunya samples in the analyzed cohorts. These genes are not arbitrary features but are directly linked to the biological processes highlighted in the enrichment analysis, including interferon signaling, immune activation, and cell-cycle regulation.

Overall, the results suggest that dengue and chikungunya infections share a common antiviral foundation driven by interferon responses but diverge substantially in their downstream transcriptional patterns. Dengue appears to be more closely associated with host cellular processes such as proliferation and DNA replication, whereas chikungunya is characterized by stronger activation of immune and inflammatory pathways. These differences provide insight into the distinct host-response profiles of the two infections and may help explain differences in their clinical manifestations.

### Limitations and Future Directions

Several limitations should be considered when interpreting the results. First, the analysis was based on a limited number of publicly available human transcriptomic datasets derived from blood samples. Although these datasets represent the most suitable available resources with well-defined healthy controls, the relatively small sample size, particularly for dengue, may reduce statistical power, increase uncertainty in feature selection, and limit generalizability. To address this concern within the available data, we added label-permutation testing and train–test gap analysis to evaluate overfitting within the cross-validation framework. These analyses supported the robustness of the observed internal classification performance, but they do not replace external validation. Accordingly, the classification results should be viewed as internal evidence of a discriminative host-response signal within the analyzed cohorts, not as clinical diagnostic validation.

Second, the available metadata and cohort design introduce important interpretive constraints. The dengue and chikungunya datasets originated from different countries and were generated from different blood-derived sample types, with dengue data obtained from peripheral blood mononuclear cells (PBMCs) in Malaysia and chikungunya data obtained from whole blood in Brazil. In addition, age, sex, symptom timing, and clinical severity were not uniformly available or directly harmonized across both datasets (Table 2). Although differential expression was performed independently within each dataset against its own healthy controls, cohort-level, demographic, geographic, sample-composition, environmental, clinical-management, and technical differences may still contribute to some observed transcriptional differences. These factors should therefore be considered when interpreting virus-specific signatures.

Third, the present study was not designed to evaluate disease progression or severity prediction. Only three severe dengue cases were available in the dengue dataset, and chikungunya chronic arthralgia follow-up information was available only for a subset of samples and was not directly comparable to dengue severity categories. Therefore, the current analysis focused on infection-status and virus-specific host-response comparison. Future studies using larger, independent, harmonized, and longitudinal cohorts will be required to validate the identified signatures across populations, sample-processing protocols, clinical settings, and prognostic applications.

## 4. Materials and Methods

### 4.1. Datasets

Two publicly available human transcriptomic datasets were used in this study to characterize host responses to dengue and chikungunya virus infections. An essential criterion for dataset selection was the availability of well-defined healthy control samples within each cohort, which enabled direct contrast between infected and non-infected individuals. This requirement ensured that differential expression analyses could be performed within each dataset under consistent biological and experimental conditions, which strengthened the identification of infection-associated gene signatures.

Dengue virus dataset (GSE279208): The DENV dataset (GSE279208) consists of bulk RNA-seq profiles derived from peripheral blood mononuclear cells (PBMCs) of adult human subjects [35]. Samples were collected from patients admitted to Hospital Kuala Lumpur, Malaysia, between January 2020 and January 2022. Dengue infection was confirmed using NS1 antigen testing, and patients were clinically classified according to World Health Organization (WHO) criteria into dengue with warning signs and severe dengue groups. Healthy individuals without prior dengue infection were included as controls. PBMCs were isolated from whole blood using Ficoll-Paque density gradient centrifugation. Total RNA was extracted using the innuPREP RNA mini kit 2.0 (Analytik Jena, Jena, Germany), followed by mRNA enrichment using Dynabeads (Invitrogen, Carlsbad, CA, USA). Sequencing libraries were prepared with the MGIEasy RNA Library Prep Set and sequenced on the DNBSEQ-G400 platform. Transcript quantification was performed using RSEM following alignment to the GRCh38 reference genome with HISAT. The dataset includes 26 samples in total, comprising 16 dengue patients and 10 healthy controls.

Chikungunya virus dataset (PRJNA507472): The CHIKV dataset (PRJNA507472) contains bulk RNA-seq profiles from peripheral whole-blood samples of adult subjects with acute chikungunya virus infection and healthy controls [12]. Patients were recruited during the 2016 outbreak in Sergipe, Brazil, and infection was confirmed by real-time RT-PCR and/or serological detection of CHIKV-specific antibodies. All samples were negative for dengue and Zika virus RNA. Blood samples were collected during the acute phase of infection, typically within the first few days following symptom onset. Total RNA was extracted from whole blood preserved in Tempus or RNAlater tubes, followed by depletion of ribosomal RNA and globin transcripts. Strand-specific libraries were generated using Illumina TruSeq protocols and sequenced on the Illumina HiSeq 1500 (Illumina, San Diego, CA, USA) platform with paired-end reads. The dataset comprises 55 samples, including 36 chikungunya-infected patients and 19 healthy controls.

Available clinical and demographic metadata for the samples analyzed in this study are summarized in Table 2. Metadata were extracted from the GEO series matrix and source publication for GSE279208, and from the source publication, Table S1, SRA RunInfo file, BioSample metadata, and preprocessing files for PRJNA507472.

It should be noted that these datasets represent the most suitable publicly available human blood-derived transcriptomic profiles for dengue and chikungunya infections with comparable study designs. While additional datasets exist, many are based on in vitro models or non-blood tissues and were therefore not compatible for inclusion in this study. The selected datasets were not fully matched by sample type, as the dengue cohort was generated from isolated PBMCs whereas the chikungunya cohort was generated from whole blood. However, because each dataset was analyzed independently for differential expression and statistical inference against its own control group, this difference did not affect the identification of infection-related gene signatures. Integrative steps such as visualization and classification were also carried out only after gene selection, normalization, and batch-effect correction. Given that the objective was to characterize circulating host transcriptional responses rather than compare unprocessed expression values directly across datasets, both cohorts were considered appropriate for inclusion.

### 4.2. Methodology

#### 4.2.1. Overall Analytical Workflow

The analytical pipeline was designed to identify robust transcriptomic signatures associated with dengue and chikungunya infections and to evaluate their potential ability to distinguish between healthy and infected samples, as well as between the two viral infections. To achieve this, the analysis was performed in five sequential stages: (i) definition of cross-validation folds within each dataset, (ii) fold-specific differential expression analysis and gene ranking performed independently in each dataset, (iii) integration of aligned folds across datasets for predictive modeling after gene selection, (iv) derivation of strict consensus gene signatures across training folds, and (v) functional enrichment analysis of the final consensus signatures. This stepwise design was adopted to preserve separation between training and test data, avoid information leakage, and support more reliable identification of candidate gene sets.

#### 4.2.2. Cross-Validation Strategy and Study Design

Because independent external validation datasets with comparable human blood-derived transcriptomic profiles were not available, internal validation was performed using a stratified *k*-fold cross-validation (CV) [17] framework with *k* = 3. Each dataset was partitioned separately into three folds, and all downstream training, feature selection, and evaluation steps were carried out within this framework (see Figure 1).

A 3-fold design was selected because the dengue cohort included only three severe dengue samples. Using three folds allowed one severe case to be represented in each fold, which improved fold balance and reduced the risk of unstable partitions. For cross-validation purposes, dengue with warning signs and severe dengue samples were grouped into a single dengue class, since the primary objective at this stage was to distinguish infected from healthy samples rather than model severity subclasses.

The dengue and chikungunya datasets were not merged during fold construction or differential expression analysis. Instead, fold assignment was performed independently within each dataset. In each iteration, two folds were used as the training subset and the remaining fold served as the independent test subset. This produced three training partitions and three matched test partitions for each virus. The strict separation between training and test sets ensured that all feature selection and model development steps were performed only on training data, while evaluation was performed on samples not used during training.

#### 4.2.3. Fold-Specific Feature Selection Within Each Dataset

Before fold-specific analysis, the expression matrices were restricted to protein-coding genes common to both datasets. This step ensured consistency in downstream comparative analyses and avoided biases related to genes that were present in only one dataset.

Feature selection was then performed independently within each training partition of each dataset. Thus, for dengue, differential expression analysis was carried out three times, once in each training fold, by contrasting dengue samples against healthy controls. The same procedure was applied independently to the chikungunya dataset by contrasting chikungunya samples against healthy controls within each training fold.

Differential expression analysis was conducted using an edgeR-based [46] framework, Generalized Linear Models with Quasi-Likelihood F-tests and Magnitude–Altitude Scoring (GLMQL-MAS) [18,19,20,21,22,23,24], which combines generalized linear modeling (GLMs) [47], quasi-likelihood F-tests [48], and Magnitude–Altitude Scoring (MAS) [25,26]. RNA-seq counts were normalized using the trimmed mean of M values method (TMM) [49], and dispersion estimates were calculated to account for biological variability. A design matrix was then used to model the contrast between infected and healthy groups within each training fold.

Statistical significance was assessed using quasi-likelihood F-tests, and *p*-values were adjusted using the Benjamini–Hochberg (BH) procedure [50]. Genes were considered significant if they satisfied both an adjusted *p*-value threshold of 0.05 and |LogFC| > 1 (i.e., LogFC > 1 or LogFC < −1). To prioritize genes according to both effect size and statistical confidence, MAS was used as a ranking measure. This produced, for each training fold and for each virus, ranked lists of significantly upregulated and downregulated genes. MAS combines both the absolute log fold change and the adjusted *p*-value:

(1) $${MAS}_{l}=|{{log}_{2}(\mathrm{FC}}_{l}){|}^{M}|{{log}_{10}(p}_{l}^{BH}){|}^{A},$$

where $p_{l}^{BH}$ denotes BH adjusted *p*-values (FDR) for *l* =1,2,…,s. Here, s is the number of rejected null hypotheses under the condition $p_{l}^{BH}<0.05$.

A larger MAS value indicates that a gene is more strongly associated with the infection condition, which reflects a combination of larger expression change and stronger statistical support. Genes were subsequently ordered in descending order of MAS values, and a rank was assigned to each gene based on this ordering. In this ranking scheme, rank 1 corresponds to the gene with the largest MAS value, while increasing rank values correspond to progressively smaller MAS scores. Therefore, there is a monotonic inverse relationship between rank and MAS score, where higher-ranked genes have higher MAS values and are considered more informative for downstream analysis.

At this stage, the two datasets corresponding to the two viruses remained completely separate (see Algorithm 1). Fold-specific differential expression analysis and ranking were performed independently within the dengue and chikungunya datasets, and no cross-dataset integration was introduced before gene selection had been completed.
**Algorithm 1.** Cross-validation, Cross-MAS feature selection, and held-out classification workflow**Input:** Dengue expression matrix and metadata; chikungunya expression matrix and metadata; common protein-coding gene set shared between the two datasets; number of folds *K = 3*; candidate numbers of selected genes per Cross-MAS category *k* ∈ {1, 2, 3, 4, 5}.
**Step 1. Define stratified folds.** Independently partition the dengue and chikungunya datasets into three folds. For dengue, assign one severe dengue case to each fold because only three severe cases were available. For classification, group dengue with warning signs and severe dengue samples into a single dengue class. For chikungunya, distribute healthy and chikungunya samples across the three folds as evenly as possible.
**Step 2. Define cross-validation iterations.** The three cross-validation iterations are:*Fold12:* folds 1 and 2 for training; fold 3 for held-out testing.*Fold13:* folds 1 and 3 for training; fold 2 for held-out testing.*Fold23:* folds 2 and 3 for training; fold 1 for held-out testing.
**Step 3. Initialize storage.** Initialize empty objects to store fold-specific ranked genes, selected feature panels, training predictions, held-out test predictions, and fold-level performance metrics.
**Step 4. Run cross-validation.** For each cross-validation iteration *i* ∈ *{Fold12, Fold13, Fold23}*:
**4.1. Define training and held-out test subsets.** Use the two folds specified by iteration *i* as the training subset and the remaining fold as the held-out test subset, separately for dengue and chikungunya.
**4.2. Perform dataset-specific differential expression and MAS ranking before cross-dataset integration.** At this stage, the dengue and chikungunya datasets remain separate with no batch-effect correction.Within the dengue training subset, compare dengue samples with dengue healthy controls using GLMQL-MAS.Within the chikungunya training subset, compare chikungunya samples with chikungunya healthy controls using GLMQL-MAS.Rank significant upregulated and downregulated genes in each dataset using the MAS score defined in Equation (1).
**4.3. Apply Cross-MAS feature integration within matched training partitions.**Match the dengue and chikungunya training partitions corresponding to iteration *i*.Apply the Cross-MAS ranking function defined in Equation (2) separately to the upregulated and downregulated ranked gene lists.Partition genes into six categories: dengue-specific upregulated, dengue-specific downregulated, chikungunya-specific upregulated, chikungunya-specific downregulated, shared upregulated, and shared downregulated.
**4.4. Select fold-specific feature panels.**For each candidate value *k* ∈ {1, 2, 3, 4, 5}, select the top *k* genes from each of the six Cross-MAS categories.Combine these genes to form the selected feature panel for iteration *i* and candidate *k*.Store the selected genes for iteration *i* and candidate *k*.
**4.5. Apply batch correction only for downstream PCA and classification.**After feature selection is complete, align the matched dengue and chikungunya training subsets to form the fold-specific training matrix for classification.Align the matched dengue and chikungunya held-out subsets to form the fold-specific held-out test matrix.Apply batch-effect correction only at this downstream modeling stage for PCA visualization and multinomial logistic regression.
**4.6. Fit PCA using training data only.**Subset the batch-corrected training expression matrix to the selected genes.Fit principal component analysis using only the training samples.Extract PC1 and PC2 from the training samples.
**4.7. Train the classifier using training data only.**Train a multinomial logistic regression model using training PC1 and PC2 as predictors.Class labels are healthy, dengue, and chikungunya.
**4.8. Evaluate the held-out test subset.**Subset the held-out test expression matrix to the same selected genes.Project held-out test samples onto the PCA axes learned from the training subset.Use the trained multinomial logistic regression model to predict held-out test labels.
**4.9. Store fold-level outputs.** Store selected genes, training predictions, held-out test predictions, balanced accuracy, macro F1, and confusion matrix results for iteration *i* and candidate *k*.
**Step 5. Aggregate outputs across folds.** After all three cross-validation iterations are completed, aggregate the stored outputs across *i* ∈ *{Fold12, Fold13, Fold23}* for each candidate value of *k*. For each *k*, combine training predictions across folds and combine held-out test predictions across folds. Calculate aggregated balanced accuracy, macro F1, and confusion matrices for training and held-out test performance. These aggregated results are used to summarize model performance across candidate feature-panel sizes.
**Output:** Fold-specific ranked gene groups, selected feature panels, training and held-out test predictions, and aggregated classification performance metrics.
**End**

#### 4.2.4. Cross-Dataset Integration and Predictive Modeling

After fold-specific gene selection had been completed independently in the two datasets, matched folds were aligned across datasets for predictive modeling. Specifically, dengue training fold 1 (DENV CV Training 1) was aligned with chikungunya training fold 1 (CHIKV CV Training 1) to form combined training set 1 (CV Training 1), and the corresponding dengue and chikungunya test folds were aligned to form combined test set 1 (CV Test 1). The same process was applied for folds 2 and 3. In this way, the two datasets were integrated only after independent feature selection within each dataset had been completed.

To enable joint modeling across studies generated using different platforms and protocols, batch-effect correction was applied after fold alignment using the *removeBatchEffect* function [51] from the *limma* package (v3.62.2). Batch correction was performed independently within each cross-validation iteration. Specifically, for each fold, the combined training set was corrected separately from the corresponding combined test set, and no expression values, batch estimates, class labels, or transformation parameters from the test samples were used during training-set correction. Likewise, the test set was corrected only within its own fold-specific test partition before projection onto the PCA space learned from the training data. This design preserved the independence of the test samples and prevented information leakage between training and test partitions.

Biological signal was preserved by applying batch correction only after infection-associated genes had been selected independently within each dataset using fold-specific differential expression analysis against the dataset’s own healthy controls. In addition, batch correction was used only for downstream PCA visualization and multinomial logistic regression, not for differential expression testing or gene ranking. The correction therefore targeted study-level technical differences between the dengue and chikungunya datasets while retaining the biological contrasts among healthy, dengue-infected, and chikungunya-infected samples that were captured by the selected gene signatures.

Within each combined training fold, the ranked fold-specific gene lists were compared across dengue and chikungunya using Cross-MAS [20]. This procedure identified six biologically meaningful gene groups: genes uniquely upregulated in dengue, uniquely downregulated in dengue, uniquely upregulated in chikungunya, uniquely downregulated in chikungunya, commonly upregulated genes, and commonly downregulated genes. Shared genes were expected to distinguish infected from healthy samples regardless of virus, whereas virus-specific genes were expected to support separation between dengue and chikungunya.

To preserve the directionality of differential expression, Cross-MAS was applied separately to upregulated and downregulated gene sets within each combined training fold. Let $r_{D}\left(g\right)$ and $r_{C}\left(g\right)$ denote the MAS-based ranks of gene g in the dengue and chikungunya datasets, respectively. For each direction, we define $G_{\mathrm{common}}=\{g|g\;\mathrm{is}\;\mathrm{significant}\;\mathrm{in}\;\mathrm{both}\;\mathrm{datasets}\}$, $G_{D}=\{g|g\;\mathrm{is}\;\mathrm{significant}\;\mathrm{only}\;\mathrm{in}\;\mathrm{dengue}\}$, and $G_{C}=\{g|g\;\mathrm{is}\;\mathrm{significant}\;\mathrm{only}\;\mathrm{in}\;\mathrm{chikungunya}\}$. The Cross-MAS ranking function $r_{\mathrm{CM}}(g)$ was then defined as

(2) $$r_{\mathrm{CM}}(g)=\{\begin{array}{cc} max\{r_{D}(g),\;r_{C}(g)\}, & g\in G_{\mathrm{common}},\\ r_{D}(g), & g\in G_{D},\\ r_{C}(g), & g\in G_{C}. \end{array}$$

For genes that were common to both datasets within a given regulation direction, the maximum of the two ranks was used to prioritize genes that were consistently highly ranked in both infections. This choice reduced the influence of genes that showed strong effects in only one dataset while favoring those that exhibited concordant behavior across both viruses. For genes unique to a single dataset, the original MAS-based rank was retained.

Finally, within each direction, genes were ordered in ascending order of $r_{\mathrm{CM}}(g)$, where rank 1 corresponds to the most informative gene under the Cross-MAS criterion. This procedure produced direction-specific ranked gene lists that were subsequently used for downstream feature selection and classification.

The resulting Cross-MAS rankings provided a unified and direction-specific prioritization of genes across the six groups, which served as the basis for downstream predictive modeling. To reduce model complexity and limit overfitting [52], only the top-*k* genes from each of the six groups were retained for classification. The optimal value of k was selected using only the training partition by evaluating classification performance across a range of candidate values and choosing the smallest value that produced optimal separation. Principal component analysis was then applied to the selected genes, and the first two principal components were used as predictors in a multinomial logistic regression model to classify samples as healthy, dengue-infected, or chikungunya-infected. Logistic regression was selected because it provides an interpretable linear framework with relatively low variance, which is advantageous in high-dimensional settings with limited sample size [52].

For each fold, the trained model was evaluated on both the training set and the independent test set, which produced three training performance estimates and three test performance estimates across the cross-validation procedure. These results were used to assess the stability and generalizability of the selected gene signatures across different data partitions. The complete cross-validation, dataset-specific gene ranking, Cross-MAS feature selection, batch-corrected PCA/classification, and held-out evaluation workflow are summarized in Algorithm 1.

To further assess potential overfitting, a label-permutation analysis was performed using the selected-gene classification workflow. For each permutation, class labels were randomly shuffled while the expression data, selected gene sets, PCA procedure, and multinomial logistic regression workflow were kept unchanged. The model was then refit using the permuted labels, and performance was evaluated on the corresponding held-out test samples to generate null distributions for test balanced accuracy and macro F1. This procedure was repeated for 1000 permutations. Empirical *p*-values were calculated as (1 + the number of permuted-label models with performance greater than or equal to the observed true-label performance) divided by (1 + the total number of permutations). In addition, the train–test performance gap was calculated for each cross-validation fold and for the aggregated analysis as training performance minus test performance for both balanced accuracy and macro F1.

#### 4.2.5. Consensus Gene Signature Identification

To identify robust gene signatures that were reproducible across data partitions, consensus analysis was performed across the three training folds. Genes were first grouped according to regulation direction and specificity, which generated separate ranked lists for dengue-specific, chikungunya-specific, and shared signatures, each considered independently for upregulated and downregulated genes.

For each category c∈{DENV-specific, CHIKV-specific, shared}×{upregulated, downregulated} and each training fold k∈{1, 2, 3}, let $G_{c}^{\left(k\right)}=\{g|g\;\mathrm{is}\;\mathrm{selected}\;\mathrm{in}\;\mathrm{fold}\;k\;\mathrm{for}\;\mathrm{category}\;c\}$ denote the set of genes obtained from the Cross-MAS ranked list, with associated ranks $r_{CM}^{\left(k\right)}(g)$ defined as in Equation (2). A strict consensus criterion was then applied within each category, such that only genes present in all three training-fold ranked lists were retained. The consensus gene set was therefore defined as the intersection

$$G_{c}^{cons}=\overset{3}{\underset{k=1}{⋂}}G_{c}^{\left(k\right)}$$

This intersection-based approach ensured that the final consensus signatures reflected genes consistently recovered across multiple training partitions rather than genes driven by a single split.

For each retained gene $g\in G_{c}^{cons}$, its rank in each training fold $r_{CM}^{\left(k\right)}(g)$ was recorded, and a conservative consensus rank was assigned as

$$r_{cons}(g)=\underset{k\in \{1,2,3\}}{max}r_{CM}^{\left(k\right)}(g)$$

This aggregation strategy prioritized genes that were not only repeatedly selected but also ranked consistently highly across all training sets, since lower (better) ranks had to be maintained across folds to achieve a favorable consensus rank.

Finally, for each category c, genes in $G_{c}^{cons}$ were ordered in ascending order of $r_{cons}(g)$, where rank 1 corresponds to the most robust gene under the consensus criterion. Consensus signatures were thus generated separately for dengue-specific, chikungunya-specific, and shared responses, and independently for upregulated and downregulated genes.

#### 4.2.6. Functional Enrichment Analysis

Functional enrichment analysis was performed on the final consensus gene signatures to characterize the biological processes and pathways associated with dengue-specific, chikungunya-specific, and shared host responses. For each category, upregulated and downregulated genes were analyzed separately to capture direction-specific pathway activity. Gene annotation was conducted using the *org.Hs.eg.db* package (v3.20.0). Enrichment analysis was carried out using *clusterProfiler* (v4.14.6) and *ReactomePA* (v1.50.0) in R (version 4.4.2). The reported results were restricted to Gene Ontology Biological Process terms, KEGG pathways, or Reactome pathways.

## 5. Conclusions

In this study, we developed an integrative transcriptomic framework that combines cross-validation-based analysis, Cross-MAS ranking, and strict consensus gene selection to investigate host responses to dengue and chikungunya infections. The results demonstrate that a small set of carefully selected genes can achieve strong classification performance, substantially improving separation between healthy, dengue, and chikungunya samples compared to models using all genes.

Beyond predictive modeling, the analysis provides clear biological insights into the similarities and differences between the two infections. Both viruses induce a shared antiviral response dominated by interferon signaling and innate immune activation, which reflects a conserved host defense mechanism. However, distinct virus-specific transcriptional programs were identified, with dengue associated with cell-cycle and DNA replication processes and chikungunya characterized by stronger activation of immune and inflammatory pathways.

The consensus gene signatures derived in this study represent reproducible features that capture these biological differences while reducing variability across data partitions. These signatures provide a compact and interpretable representation of host transcriptional responses and may serve as candidate host-response markers for distinguishing infection-associated transcriptional states after validation in independent cohorts.

Overall, this work highlights the value of integrating feature selection, predictive modeling, overfitting assessment, and biological interpretation in transcriptomic studies. The proposed framework can be extended to other infectious diseases and may contribute to future host-response biomarker studies and a deeper understanding of host–pathogen interactions.

## Figures and Tables

**Table 1:** Biological context for representative consensus genes. Representative genes were selected from the consensus signatures. The table summarizes the signature category in this study and previously reported biological functions or infection-related relevance. These annotations were used for biological interpretation only and were not used for feature selection or model training.

Genes	Category	Biological Relevance
*USP18*, *ISG15*	Shared upregulated	Interferon-stimulated antiviral-response genes. *USP18* is a multifunctional component of interferon response and regulates ISG15-dependent pathways; *ISG15* and ISGylation are broadly involved in innate antiviral immunity [27,28].
*IFI27*, *IFI6*	Shared upregulated	Interferon-stimulated genes consistent with antiviral innate immune activation [29,30]. *IFI27* has been reported as highly expressed in dengue transcriptomic studies and linked to interferon/JAK-STAT-associated responses [30].
*CXCL10*, *CCL2*	Shared upregulated	Chemokines involved in leukocyte recruitment and inflammatory antiviral responses. *CXCL10* and *CCL2* have been reported in Chikungunya inflammatory signatures [31,32].
*SDC1*, *MZB1*	DENV-specific upregulated	*SDC1*/*syndecan-1* has been associated with endothelial glycocalyx disruption and plasma leakage in dengue [33]. *MZB1* is linked to B-cell and plasmablast biology, which is consistent with dengue-associated humoral immune activation [34,35].
*BUB1*, *RRM2*	DENV-specific upregulated	Cell-cycle and DNA-synthesis-associated genes. A prior dengue transcriptomic analysis reported *BUB1* and *RRM2* among genes upregulated in dengue and severe dengue patients [36], and broader studies show that dengue and other viral infections can alter host cell-cycle or replication-associated pathways [37,38].
*TNFAIP3*, *ATF3*	CHIKV-specific upregulated	Immune-regulatory and stress-response genes. *TNFAIP3*/*A20* regulates inflammatory signaling and cell-death pathways during viral infection, while *ATF3* is linked to integrated stress-response and innate immune regulation during viral infection [39,40,41].
*GPR84*, *PLAU*, *SERPING1*	CHIKV-specific upregulated	Genes linked to inflammatory, myeloid/macrophage, proteolytic, and complement-related processes. *GPR84* enhances inflammatory signaling in macrophages, and *SERPING1* encodes C1 inhibitor, a regulator of complement activation [12,42].

**Table 2:** Metadata summary of the analyzed dengue and chikungunya datasets. Metadata availability differed between datasets, and not all clinical or demographic variables were available at the same resolution across cohorts.

Metadata Item	Dengue Dataset, GSE279208	Chikungunya Dataset, PRJNA507472
Disease group analyzed	Dengue virus infection versus healthy controls	Chikungunya virus infection versus healthy controls
Study location	Malaysia	Brazil
Sample source	Peripheral blood mononuclear cells	Whole blood
Samples analyzed	26 total: 16 dengue and 10 healthy controls	55 total: 36 chikungunya and 19 healthy controls
Age information	Available as group-level summaries from the source paper: healthy controls, median 31 years, Interquartile Range (IQR) 23 to 35; dengue with warning signs, median 23 years, IQR 18 to 33; severe dengue, 31 years, IQR 21 to 52	Available as individual-level BioSample metadata: chikungunya, median 27 years, IQR 22 to 37.25, range 16 to 79; healthy controls, median 53 years, IQR 38 to 59, range 24 to 76
Sex information	Not available in the public GEO series matrix or source-paper summary tables	Available from SRA/BioSample metadata: chikungunya, 24 female and 12 male; healthy controls, 14 female and 5 male
Symptom timing	Available as group-level days of fever from the source paper: dengue with warning signs, median 5 days, IQR 4 to 5.5; severe dengue, 6 days	Available from the source study Table S1 after matching: 21 at day 0 or 1, 7 at day 2, 7 at days 3 or 4, and 1 at day 20 or later after symptom onset
Diagnostic confirmation	Dengue NS1-positive; cases classified according to World Health Organization criteria	CHIKV real-time RT-PCR and/or CHIKV-specific IgM (as determined by serodiagnosis ELISA); all samples were negative for dengue and Zika virus RNA

## Data Availability

The RNA sequencing datasets analyzed in this study are publicly available through the NCBI Gene Expression Omnibus and the NCBI Sequence Read Archive. The dengue dataset is accessible under accession number GSE279208, and the chikungunya dataset is available under BioProject accession PRJNA507472. All data were generated by the original study authors and are used in this study in accordance with their open access availability.

## Supplementary Materials

The following supporting information can be downloaded at: https://www.mdpi.com/article/10.3390/ijms27125552/s1.

## Abbreviations

The following abbreviations are used in this manuscript:

BH	Benjamini–Hochberg
CHIKV	Chikungunya virus
COVID-19	Coronavirus disease 2019
CV	Cross-validation
DENV	Dengue virus
DE	Differential expression
FDR	False discovery rate
GLM	Generalized linear model
GLMQL-MAS	Generalized Linear Models with Quasi-Likelihood F-tests and Magnitude–Altitude Scoring
GO BP	Gene Ontology Biological Process
KEGG	Kyoto Encyclopedia of Genes and Genomes
log_2_FC	Log_2_ fold change
MAS	Magnitude–Altitude Scoring
OTE	Organ tissue equivalent
PBMC	Peripheral blood mononuclear cell
PC	Principal component
PCA	Principal component analysis
QL	Quasi-likelihood
RNA-seq	RNA sequencing
RT-PCR	Reverse transcription polymerase chain reaction
TMM	Trimmed mean of M values
WHO	World Health Organization
